# The CDC Hemophilia B mutation project mutation list: a new online resource

**DOI:** 10.1002/mgg3.30

**Published:** 2013-08-19

**Authors:** Tengguo Li, Connie H Miller, Amanda B Payne, W Craig Hooper

**Affiliations:** Division of Blood Disorders, National Center on Birth Defects and Developmental Disabilities, Centers for Disease Control and PreventionAtlanta, Georgia

**Keywords:** F9 gene, hemophilia B, mutation database.

## Abstract

Hemophilia B (HB) is caused by mutations in the human gene *F9*. The mutation type plays a pivotal role in genetic counseling and prediction of inhibitor development. To help the HB community understand the molecular etiology of HB, we have developed a listing of all *F9* mutations that are reported to cause HB based on the literature and existing databases. The Centers for Disease Control and Prevention (CDC) Hemophilia B Mutation Project (CHBMP) mutation list is compiled in an easily accessible format of Microsoft Excel and contains 1083 unique mutations that are reported to cause HB. Each mutation is identified using Human Genome Variation Society (HGVS) nomenclature standards. The mutation types and the predicted changes in amino acids, if applicable, are also provided. Related information including the location of mutation, severity of HB, the presence of inhibitor, and original publication reference are listed as well. Therefore, our mutation list provides an easily accessible resource for genetic counselors and HB researchers to predict inhibitors. The CHBMP mutation list is freely accessible at http://www.cdc.gov/hemophiliamutations.

## Introduction

Hemophilia is a disorder characterized by defective blood clotting, which is classified as Hemophilia A or B and is X-linked recessive, affecting primarily males (Bowen 2002). Hemophilia B (HB), also known as Christmas disease, is caused by mutations in the gene *F9*, which is located at the distal end of the long arm of the X chromosome (Yoshitake et al. 1985). *F9* encodes coagulation factor IX (FIX), a serine protease functioning as a blood clotting factor, which is mainly expressed in liver cells (Anson et al. 1984). The gene contains eight exons and spans 33.5 kb. The 2.8-kb mRNA encodes a single-chain precursor protein of 461 amino acids, which undergoes a series of intracellular posttranslational modifications, including hydroxylation and γ-carboxylation (Anson et al. 1984; Roberts 1993; Yoshitake et al. 1985). During the process of secretion, the N-terminal signal peptide and the propeptide region are removed through proteolytic cleavage, and the protein is secreted as an inactive zymogen (Bentley et al. 1986). Upon the activation by factor XIa of the intrinsic coagulation pathway or by the tissue factor/FVIIa complex of the extrinsic pathway, the activation peptide in the middle of the protein (residues 146–180) is cleaved out to give rise to a mature protein, an active serine protease (FIXa) composed of a light chain and a heavy chain linked by a single disulfide bond (Kurachi and Davie 1982; Taran 1997). The light chain comprises three structural domains: the Gla domain and two epidermal growth factor-like domains. The heavy chain consists of one domain – the serine protease domain. FIXa participates in the blood clotting process through cleavage of an arginine–isoleucine bond of factor X to form activated factor X (FXa) in the coagulation cascades (Zhong et al. 2002). Hence, functionally defective or reduced FIX in the blood stream resulting from mutations in *F9* leads to impaired blood clotting. Typically, HB patients have prolonged coagulation time and exhibit trauma and surgery induced, and if more severe, spontaneous bleeding. The symptoms of HB vary along a spectrum from mild to severe. Normal FIX plasma activities range from 50 to 150%. Patients with less than 1% of the normal activity are classified as severe. Those with 1–5% FIX activity are moderate, whereas those who have >5 to <40% FIX activity fall into the mild category (White et al. 2001).

HB occurs at a frequency of one in 25,000–30,000 male births, which is about five times rarer than Hemophilia A (Bolton-Maggs and Pasi 2003; Giannelli et al. 1998). A wide spectrum of mutations that cause HB have been identified in all regions of *F9*, including the promoter and 3′ untranslated region (3′UTR). These mutations include: point mutations, gross deletions, frameshifts caused by insertions and small deletions, and gene regulation and splicing defects (Giannelli et al. 1998; Miller et al. 2012). Unlike hemophilia A, no inversions have been reported in HB. Point mutations account for the vast majority of HB cases, causing changes in amino acids (missense) or creating a stop codon resulting in premature stop of translation (nonsense). A point mutation in the gene regulation region may change the gene expression levels. For example, in HB Leyden, the plasma FIX levels are less than 1% of normal in childhood but increase to up to 70% of normal after puberty (Briet et al. 1982). The mechanism underlying this is the impairment of transcription factor hepatocyte nuclear factor 4 binding to androgen receptors. As a result, constitutive expression of FIX is disrupted. Several promoter mutations, such as c.−49T>A and c.−50T>G, have been identified to be associated with this phenotype (Reijnen et al. 1993). The documentation of *F9* mutations and the associated phenotypes is important for validation and study of recurrent and novel mutations.

Databases documenting *F9* mutations, such as the FIX mutation database (http://www.factorix.org/), the human gene mutation database (HGMD) (http://www.hgmd.cf.ac.uk/), and UniProt (http://www.uniprot.org/), provide web-based search for mutations underlying HB. Although these databases offer useful information to identify recurrent mutations, they are not straightforward or comprehensive. We sought to develop a simple, straightforward, and comprehensive mutation list with relevant information in a single file that is easy to search. In this report, we describe the Centers for Disease Control and Prevention (CDC) Hemophilia B Mutation Project (CHBMP) mutation list containing more than 1000 unique mutations. The new mutation list attempts to serve as a complement to the existing databases to provide a resource to the HB community for genetic counseling and inhibitor prediction. Similar to the CDC Hemophilia A Mutation Project (CHAMP) (Payne et al. 2013), in CHBMP, mutations in *F9* are documented according to standard nomenclature, classified based on the nature and positions of the molecular defects, and linked to relevant phenotypes. The CHBMP mutation list is presented in a Microsoft Excel file and is a free resource that can be accessed and downloaded from the CDC website at: http://www.cdc.gov/hemophiliamutations (also see supplemental file). The databases will be updated quarterly to included recently published or submitted novel mutations in *F9* that cause HB.

## Material and Methods

### Data collection

The data listed in the CHBMP mutation list were collected from the current *F9* mutation databases combined with a systematic literature search. The mutations in existing public access *F9* mutation databases including the FIX mutation database (http://www.factorix.org/), its previous version, HGMD (http://www.hgmd.cf.ac.uk/), and UniProt (http://www.uniprot.org/) were identified. The cited publications for each mutation in these databases were reviewed for accuracy. A systematic literature search using PubMed (http://www.ncbi.nlm.nih.gov/pubmed) was conducted for publications that reported *F9* mutations not listed in the current databases using the following terms: “Hemophilia B and mutation,” “F9 and mutation,” and “factor 9 and mutation.” Finally, mutations that were identified in the CDC laboratory as part of the Hemophilia Inhibitor Research Study (HIRS) (Miller et al. 2012) that were not published were added. Quarterly PubMed searches will be conducted to identify novel mutations for CHBMP updates.

### Data extraction and preparation

Data were compiled as described in the CDC CHAMP mutation list (Payne et al. 2013). Briefly, only single mutations that potentially cause HB were included in the CHBMP mutation list. To avoid phenotypic ambiguity, mutations in female carriers and patients carrying multiple disease-causing mutations were excluded. Similarly, polymorphisms were excluded because they are not associated with the HB phenotype. Each mutation was assigned a name for cDNA according to the nomenclature recommendations of the HGVS (http://www.hgvs.org/mutnomen/) based on the reference sequence NM_000133.3. The corresponding positions of the mutations in the human genome version hg19 and in the widely used nucleotide sequence described in Yoshitake et al. (1985) were also provided. When available, predicted changes in amino acids caused by the mutations were named using HGVS nomenclature based on protein reference sequence NP_000124.1. The widely used mature protein nomenclature, in which the numbering started at the first amino acid of the processed protein, was listed as well (Yoshitake et al. 1985). Each mutation was also provided with additional information including: the exonic, codon and domain location of the mutation; the mutation type and the underlying mechanism; the disease severity associated with each mutation; the inhibitor history; and the reference that first reported the mutation and the year published.

As described in Payne et al. (2013), the mutation types were classified into nine categories: missense, nonsense, frameshift, synonymous, promoter, 3′UTR, small structural changes (in frame; <50 bp), splice site change, and large structural changes (>50 bp). The molecular defects resulting in these mutation types were listed under Mechanism. These included: substitution, deletion, duplication, insertion, and deletion/insertion, if a number of base pairs were deleted and extra base pairs were inserted in the same place in *F9*. Exon and codon identify the exons where the mutation is located and codons the mutation affects, respectively. The domains, which were defined based on Chavali et al. (2011), identify the domain locations of the mutation. The subtype of each mutation indicates the mutation locations in one of the following: promoter, signal peptide, propeptide, light chain, heavy chain, and 3′UTR.

According to the recommendation of the Scientific and Standardization Committees of the International Society of Thrombosis and Haemostasis (ISTH) (White et al. 2001), the severity of each mutation was classified based on FIX activity levels as severe (<1%), moderate (1–5%), and mild (>5 to <40%). Reported severity listed the severity reported in the original articles.

The history of inhibitors of each mutation was listed if this was reported from any of the following sources: the original publication, the current HB database, or record in the CDC HIRS database. Otherwise, Not Reported was listed. Each mutation was provided with a reference that first reported the mutation. If a mutation was not published in the literature but was in the FIX mutation database (http://www.factorix.org/) or was identified in HIRS, it was cited as FIX mutation databases or CDC, respectively. The year of the publication was listed in a separate field. Additional information such as potential functional consequences of the mutation, whether or not the mutation occurs at a CpG site, and the dbSNP polymorphism number, if applicable, were listed under “Comments.”

### *F9* mutation list design and implementation

The CHBMP mutation list is developed in the format of a Microsoft Excel file. There are eight tabs in the Excel workbook. Table of Contents summarizes the contents of each tab. The CHBMP mutation list tab contains all the information of *F9* mutations described above. The Field Definition tab provides explanations for the fields that are used to describe each mutation. There are six figures in the Figures tab to summarize the data in the CHBMP mutation list. The Tables tab contains two tables that summarize the frequency of mutation types of *F9*, inhibitor information, and the associated phenotypes. All the references cited in the CHBMP mutation list are listed in the References tab. A search guide is provided in the Instructions tab. A form for mutation submission and correction is provided in the Submission tab which allows users to provide information for updates and corrections.

## Results and Discussion

**Figure 1:**
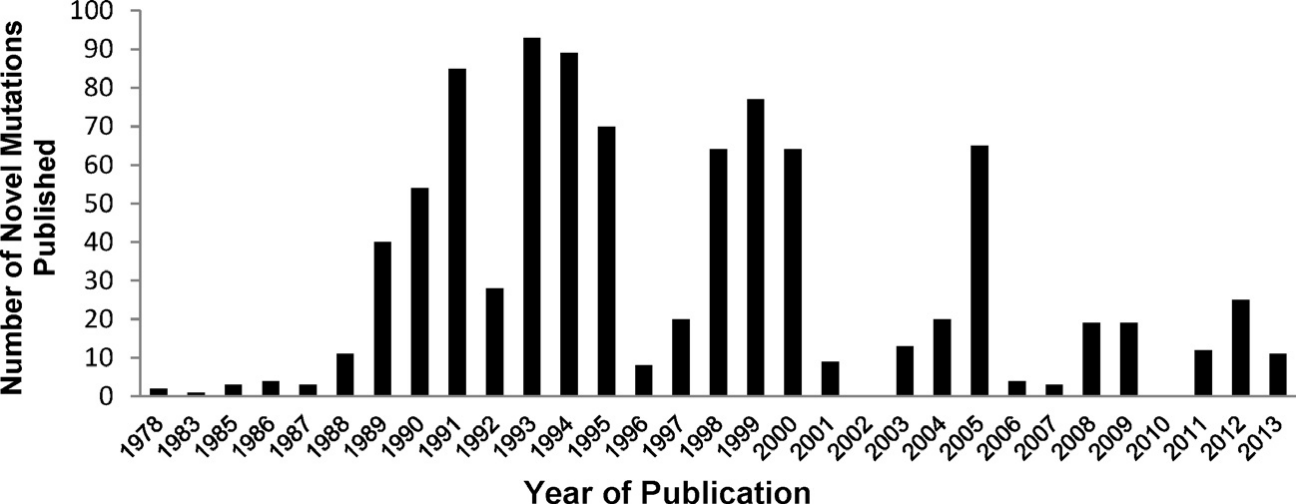
Novel F9 mutations by year of publication.

The CHBMP mutation list contained 1083 unique mutations as of March 2013. Only mutations reported to cause HB phenotypes are included. Other polymorphisms, mutations in female carries, and mutations in patients with multiple mutations in *F9* were excluded due to the possible ambiguous association between mutations and phenotypic severity. These mutations were identified from more than 3700 HB cases reported within the past decades. Figure 1 shows the number of novel mutations reported by year of publication. The first mutation was identified in 1978. A few more novel mutations were identified in the early 1980s. The majority of the mutations were identified during the 1990s. More novel mutations have been continually identified since then.

### Mutation frequency and distribution over *F9*

The frequencies of the nine mutation types are outlined in Table 1. The most common mutation in *F9* was missense, which accounted for 58.4%. A smaller percentage (15.4%) of the mutations was frameshifts caused by deletion, duplication, or insertion. Nonsense (8.3%) and splice site (9.4%) mutations were common in the population as well. Other mutations, large structural change (3.0%), small structural change (1.9%), synonymous mutation (0.9%), and mutations in promoter (2.2%), or 3′UTR (0.4%) accounted for a small percentages of listed mutations.

**Table 1:** Frequencies of *F9* mutation type and mechanisms and number of reported inhibitors in CHBMP database

Mutation type	Mechanism	No. of mutations	% of mutations	No. of mutations reported with inhibitors
Missense	All	633	58.4	2
	Substitution	630	58.2	1
	Insertion/Deletion	3	0.3	1
Nonsense	Substitution	90	8.3	6
Frameshift	All	167	15.4	6
	Deletion	125	11.5	5
	Duplication	3	0.3	0
	Insertion	31	2.9	0
	Insertion/Deletion	8	0.7	1
Splice site change	All	102	9.4	1
	Substitution	86	7.9	1
	Deletion	14	1.3	0
	Insertion	2	0.2	0
Large structural change (>50 bp)	All	33	3.0	9
	Deletion	29	2.7	9
	Insertion	4	0.4	0
Small structural change (in frame, <50 bp)	All	21	1.9	0
	Deletion	17	1.6	0
	Duplication	1	0.1	0
	Insertion	1	0.1	0
	Insertion/Deletion	2	0.2	0
Synonymous	Substitution	10	0.9	0
Promoter	All	23	2.1	0
	Substitution	21	1.9	0
	Deletion	2	0.2	0
3′UTR	Substitution	4	0.4	0
Total		1083	100.0	24

CHBMP, Centers for Disease Control and Prevention (CDC) Hemophilia B Mutation Project.

The development of neutralizing antibodies, termed inhibitors, has been reported in 1.4–11.7% of HB patients and in 3.7–24% of severe cases (Radic et al. 2013). It has also been reported that more than 20% of patients with gross deletions develop inhibitors (DiMichele 2007). For most of the mutations in the mutation list, inhibitor status was not reported in the original publications. If inhibitors were reported in any patient carrying a mutation, the mutation was counted as being associated with inhibitors. Only 136 (12.6%) mutations had inhibitor status reported. Twenty four of them, including nine large deletion, two missense, six nonsense, six frameshift, and one splice mutations, were associated with inhibitors.

**Figure 2:**
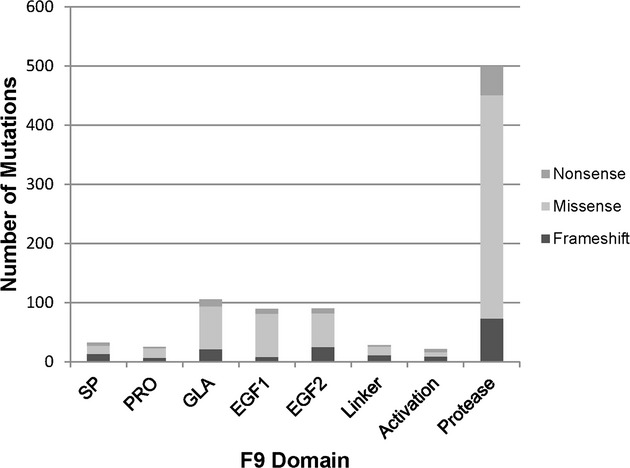
Frequencies of missense, nonsense, and frameshift mutations by domain.

**Figure 3:**
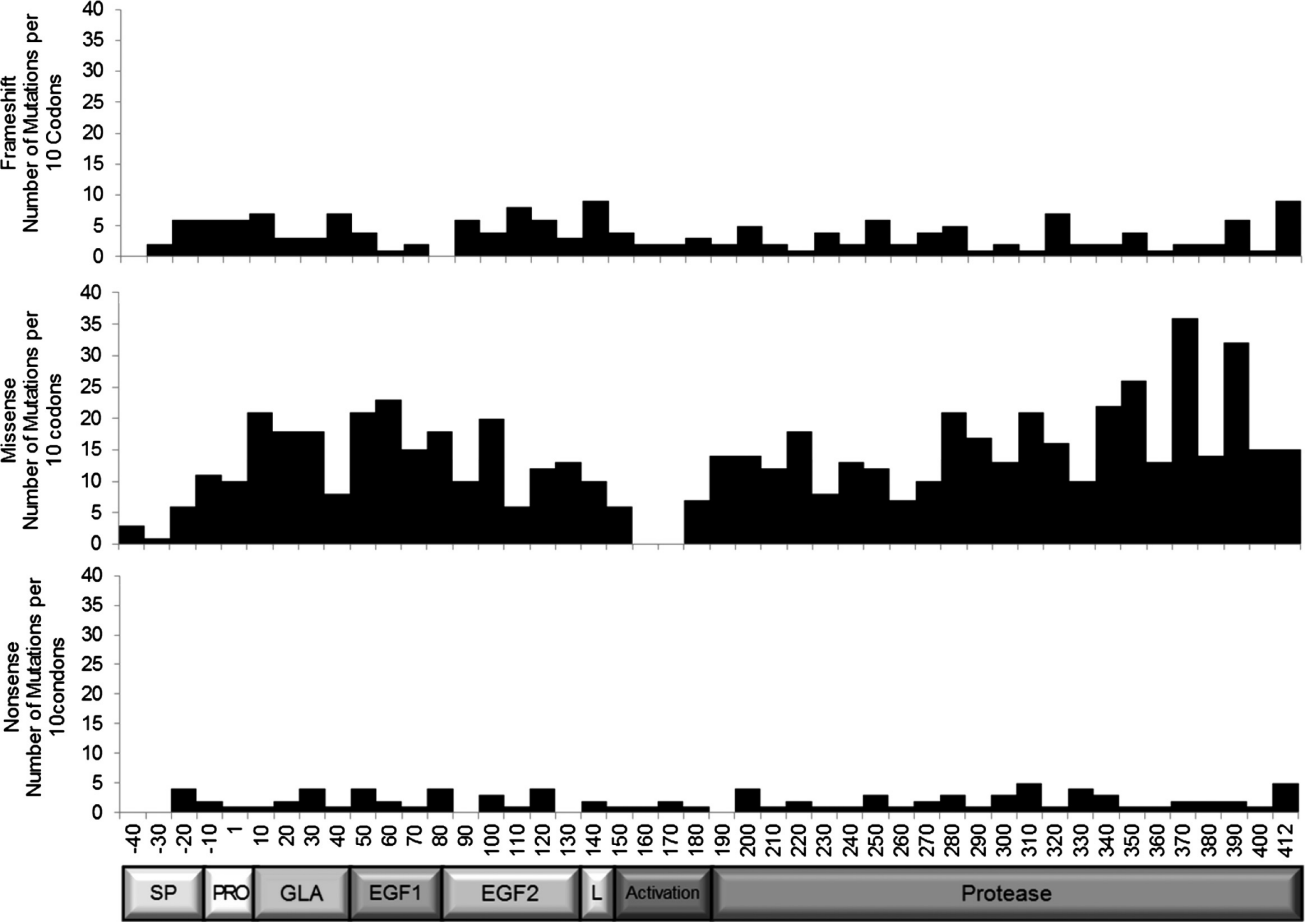
Distribution of missense, nonsense, and frameshift mutations in *F9* by codon.

Figure 2 shows the distribution of frameshift, missense, and nonsense mutations throughout the FIX protein. All three types of mutations are represented in every domain of FIX; however, the majority of the mutations are located in the protease domain (Fig. 2). The map of the mutations on FIX according to mutation type is shown in Figure 3 with each bar representing the number of mutations per 10 codons. Frameshift and nonsense mutations are evenly distributed throughout the FIX protein. This is expected as these types of mutations may cause premature stop of translation, thus leading to defective or truncated proteins regardless of their location. Missense mutations are present in all domains except the middle region of the activation domain. Consistent with previous reports (Giannelli et al. 1998), signal peptide and activation domains are underrepresented among missense mutations due to the lack of importance of most of their amino acids. Because the activation domain is cleaved off during the process of activation, it is less functionally important. However, missense mutations are found in the junction regions between the activation domain and its adjacent domains suggesting that the mutation might interfere with the normal cleavage process and result in the production of dysfunctional FIX proteins.

**Figure 4:**
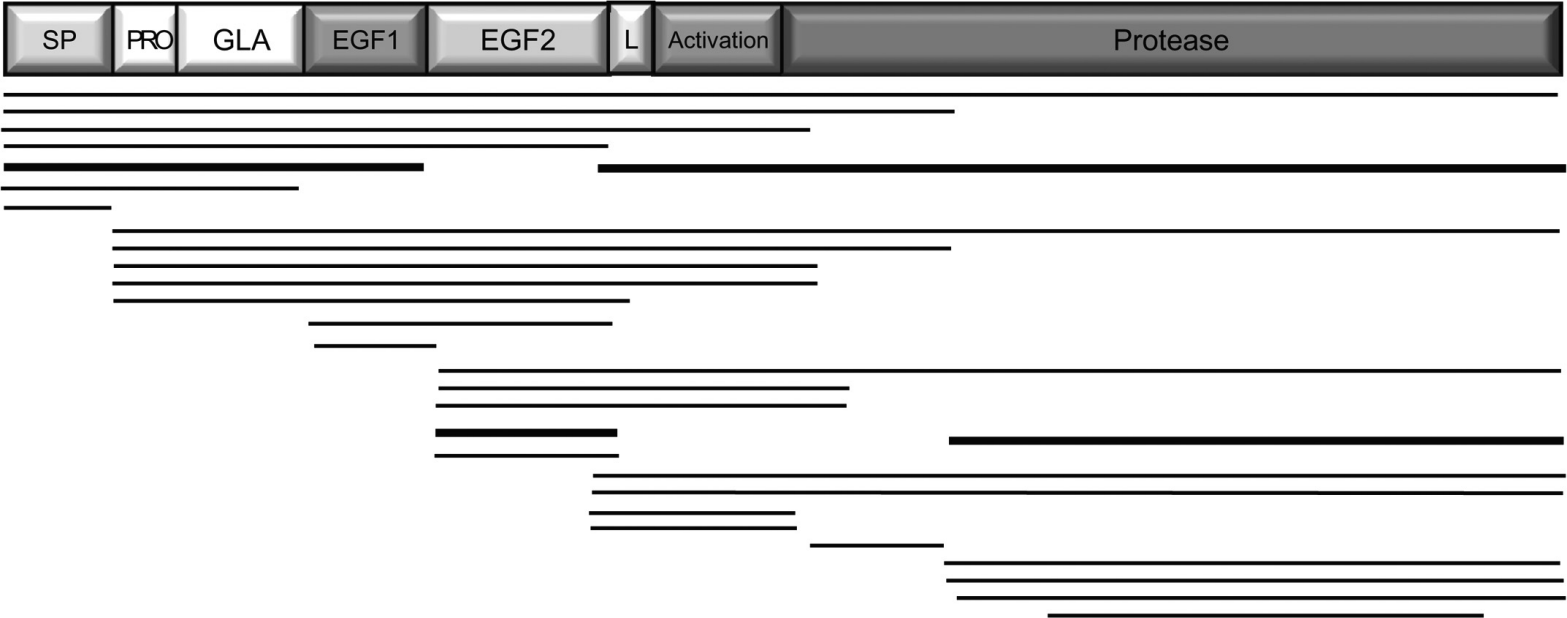
Reported large deletions in F9: lines indicate the deleted regions and do not represent the number of mutations. Bold lines at same level indicate two deletions in one patient.

Large structural changes, including deletions and insertions involving more than 50 bp, often have deleterious effect and are associated with severe phenotype. There are 33 different deletions and insertions in the CHBMP mutation list, 29 of which are large deletions ranging from single-domain deletions to whole-gene deletions. Notably, there are two large deletions spanning two noncontinuous regions of FIX (Fig. 4). One issue is that the exact break points for the majority of large deletion mutations are not reported in the literature. We have distinguished them at the exon level. Therefore, the true number of unique large deletions is likely greater than that in our list.

### Mutations and severity

The severity reported in the original publication is listed in a separate column in the CHBMP mutation list. Some of the mutation types exhibited variability in severity among different patients having the same mutation type. This is due to the fact that severities were classified in some publications using a different standard than that currently adopted by the ISTH. Here, we summarize the distribution of severity for the mutation types based on the ISTH standard (Table 2). The severity was reported for 902 mutations (83.3%) of the total 1083 mutations listed. A total of 194 mutations (21.5%) were reported with inconsistent severities. The majority of the mutations (442, 49%) were associated with the severe phenotype only. This is the case for most of the mutation types except mutations in the promoter and 3′UTR. The moderate phenotype only was reported for 147 mutations (16.3%), and the mild phenotype only for 119 mutations (13.2%). The vast majority of frameshift (78.5%), nonsense (75.3%), and large structural change mutations (90%) were associated with the severe phenotype. This is expected as these mutations result in fundamental changes in the FIX protein. Mutations in the promoter may affect the transcription efficiency and result in a low level of FIX as well as the Leyden phenotype. Only 16.7% of the mutations in the promoter led to severe phenotype. None of the mutations in 3′UTR caused severe HB (Table 2).

**Table 2:** Severity (based on ISTH standard) and mutation types

Mutation type	Total	Severe (%)	Moderate (%)	Mild (%)	Various severities (%)
Missense	558	218 (39.1)	98 (17.6)	100 (17.9)	142 (25.4)
Nonsense	77	58 (75.3)	2 (2.6)	1 (1.3)	16 (20.8)
Frameshift	130	102 (78.5)	20 (15.4)	3 (2.3)	3 (3.8)
Splice site change	83	41 (49.4)	18 (21.7)	6 (7.2)	18 (21.7)
Large structural change (>50 bp)	10	9 (90)	1 (10)	0	0
Small structural change (in frame, <50 bp)	15	11 (73.3)	3 (20)	0	1 (6.7)
Synonymous	7	0	0	3 (42.9)	4 (57.1)
Promoter	18	3 (16.7)	4 (22.2)	5 (27.8)	6 (33.3)
3′UTR	4	0	1 (25.0)	1 (25.0)	2 (50.0)
Total	902	442 (49.0)	147 (16.3)	119 (13.2)	194 (21.5)

ISTH, International Society of Thrombosis and Haemostasis.

**Figure 5:**
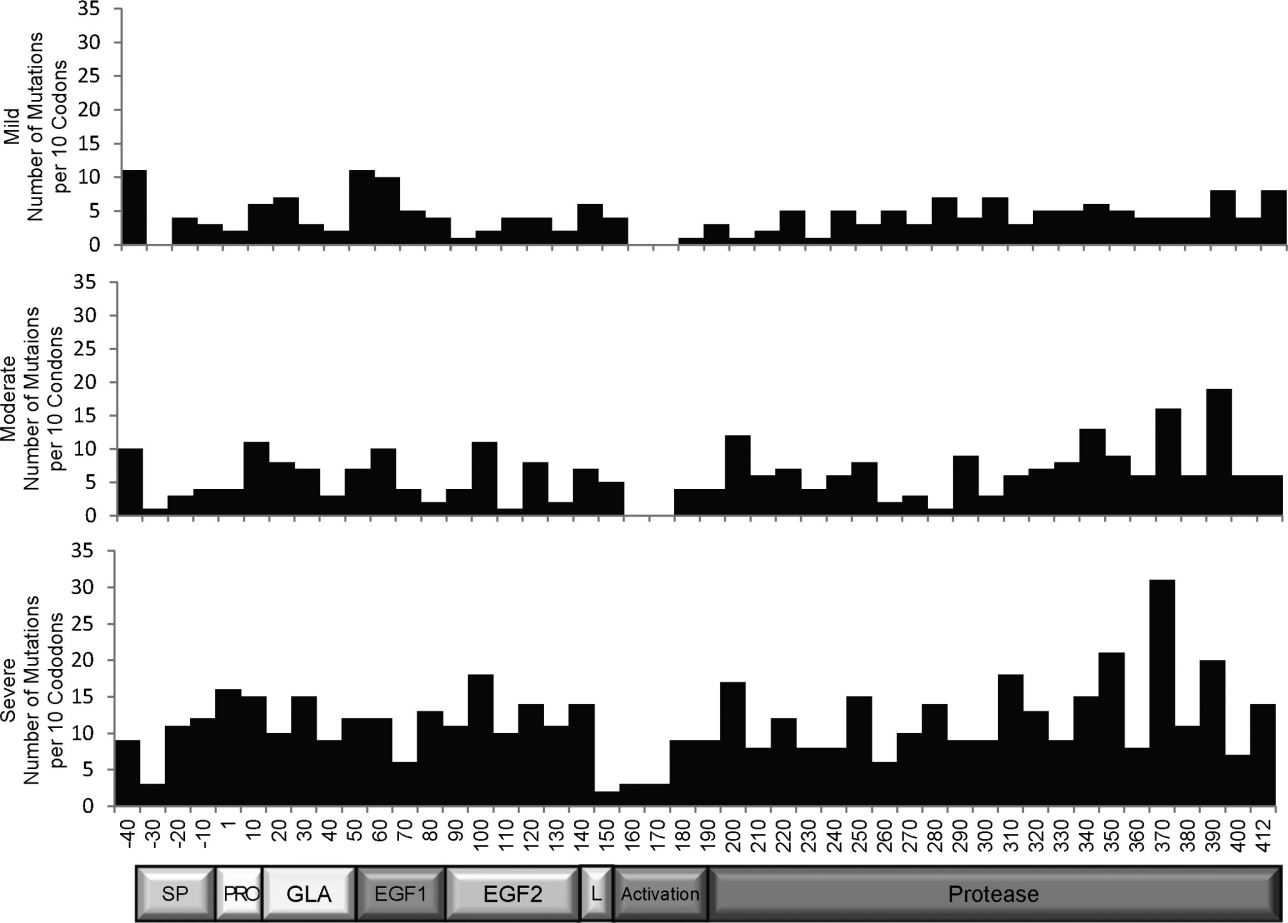
Distribution of mutations associated with mild, moderate, and severe Hemophilia B throughout *F9*. Classes of mutations include missense, frameshift, nonsense, promoter, and synonymous mutations.

The mutations associated with mild, moderate, and severe phenotypes are distributed evenly throughout *F9* (Fig. 5). The distribution patterns are similar among the three categories of phenotypic severity. Mutations associated with severe phenotype account for the majority in all domains of FIX. It was noted that neither mild nor moderate phenotype was reported within a small part of the activation domain. This is likely due to the fact that the domain is cleaved in the course of activation. Mutations identified in this region were either frameshift or nonsense, which mainly causes severe phenotype.

## Conclusions

In this study, we describe the CHBMP mutation list, which contains more than 1000 unique mutations that are reported to cause HB. Like CHAMP, CHBMP is not meant to replace other current databases but to provide a complement. It was designed to help the hemophilia community to better understand the molecular mechanisms underlying the disease and to foster collaboration. Bearing this in mind, we have developed a comprehensive but straightforward mutation list for *F9* that is freely accessible. This new resource attempts to integrate all the related information such as mutation types and phenotypic severity into one single Microsoft Excel file that allows researchers to easily search, analyze, and compile the mutations associated with HB. The mutations are listed with standard and commonly used nomenclature and classified into various mutation types. The resource also contains information regarding predicted mechanisms that cause changes in *F9* expression, mRNA processing, and protein structure. The disease severity and inhibitor development associated with each mutation are included as well. Thus, our mutation list will provide a complete, easy to use resource for HB researchers and clinicians to identify novel and recurrent mutations.

## Supporting Information

**Figure S1.** Number of novel F9 mutations by year of publication.

**Figure S2.** Frequency of F9 mutation types in the CHBMP mutation list.

**Figure S3.** Number of mutation type by FIX domain.

**Figure S4.** Distribution of missense, nonsense and frameshift mutations in F9 by codon.

**Figure S5.** Large deletions in F9 in the CHBMP mutation list.

**Figure S6.** Distribution of F9 mutations associated with mild, moderate and severe HemB.
